# Insulin Use and Clinical Outcomes in Patients Undergoing Coronary Artery Bypass Graft Surgery

**DOI:** 10.21470/1678-9741-2019-0347

**Published:** 2020

**Authors:** David N. Ranney, Judson B. Williams, Álvaro S. Albrecht, Shuang Li, Renato A. K. Kalil, Eric D. Peterson, Renato D. Lopes

**Affiliations:** 1 Duke Clinical Research Institute, Durham, North Carolina, United States of America.; 2 Duke University School of Medicine, North Carolina, United States of America.; 3 Instituto de Cardiologia do Rio Grande do Sul/Fundação Universitária de Cardiologia, Santana, Porto Alegre, Rio Grande do Sul, Brazil.; 4 Department of Cardiology, Universidade Federal de Ciências da Saúde de Porto Alegre, Porto Alegre, Rio Grande do Sul, Brazil.; 5 Universidade Federal de São Paulo, São Paulo, São Paulo, Brazil.

**Keywords:** Insulin, Regular, Human, Coronary Artery Bypass, Intensive Care Units, Operating Rooms, Blood Glucose, Logistic Models

## Abstract

**Objective:**

To describe insulin use and postoperative glucose control in patients undergoing coronary artery bypass graft (CABG) surgery.

**Methods:**

We examined 2,390 patients with and without diabetes enrolled in the Contemporary Analysis of Perioperative Cardiovascular Surgical Care (CAPS-Care) Study who underwent CABG surgery (01/2004 - 06/2005) to describe postoperative insulin use, variation in insulin use across different hospitals, and associated in-hospital complications and clinical outcomes. Logistic regression was used to assess the adjusted relationship between insulin use and clinical outcomes.

**Results:**

Overall, insulin was used in 82% (n=1,959) of patients, including 95% (n=1,203) with diabetes (n=1,258) and 67% (n=756) without diabetes (n=1,132). Continuous insulin was used in 35.5% of patients in the operating room and in 56% in the intensive care unit. Continuous insulin use varied significantly among centers from 8-100% in patients with diabetes. When compared with all patients not receiving insulin, insulin use in patients without diabetes was associated with a higher rate of death or major complication (adjusted odds ratio [OR]=1.54; 95% confidence interval [CI] 1.15-2.04; *P*=0.003). In patients with diabetes, insulin use was not associated with a higher risk of adverse outcomes (adjusted OR=1.01; 95% CI 0.52-1.98; *P*=0.98).

**Conclusion:**

The postoperative use of insulin is high among CABG patients in the United States of America. Insulin use in patients without diabetes was associated with worse clinical outcomes compared to patients (both with and without diabetes) who did not receive insulin. Further investigation is needed to determine the optimal use of postoperative insulin after CABG.

**Table:** 

Abbreviations, acronyms & symbols				
**ACSD**	**= Adult Cardiac Surgery Database**	** **	**ESRD**	**= End-stage renal disease**
**AI**	**= Aortic insufficiency**		**IABP**	**= Intra-aortic balloon pump**
**AS**	**= Aortic stenosis**		**ICU**	**= Intensive care unit**
**AVR**	**= Aortic valve replacement**		**MI**	**= Myocardial infarction**
**BMI**	**= Body mass index**		**MR**	**= Mitral regurgitation**
**CABG**	**= Coronary artery bypass graft**		**MS**	**= Mitral stenosis**
**CAD**	**= Coronary artery disease**		**MVr**	**= Mitral valve repair**
**CAPS-Care**	**= Contemporary Analysis of Perioperative Cardiovascular Surgical Care**		**MVR**	**= Mitral valve replacement**
**CHF**	**= Congestive heart failure**		**NYHA**	**= New York Heart Association**
**CI**	**= Confidence interval**		**OR**	**= Odds ratio**
**CVA**	**= Cerebrovascular accident**		**STS**	**= Society of Thoracic Surgeons**
**EF**	**= Ejection fraction**		**VF**	**= Ventricular fibrillation**
**eGFR**	**= Estimated glomerular filtration rate**		**VT**	**= Ventricular tachycardia**

## INTRODUCTION

Coronary artery bypass graft (CABG) surgery continues to provide a survival advantage over initial medical therapy for many groups of patients, including those with diabetes mellitus^[1]^. As the proportion of patients with diabetes undergoing CABG continues to increase (33% in the year 2000 to 40% in 2009) and in order to maintain favorable clinical outcomes, attention to perioperative glycemic control in the intensive care unit (ICU) has become increasingly important^[2]^.

In 2001, a study from Van den Berghe et al.^[3]^ at a Belgian hospital introduced the concept and importance of tight glycemic control in the ICU; results from this study influenced the recommendations of several medical societies^[4-6]^. Nevertheless, subsequent studies have failed to reproduce these findings, thereby challenging the initial results of the Belgian hospital study^[3]^. Current data demonstrate that treatment of hyperglycemia with insulin during CABG surgery can lead to postoperative hypoglycemia^[7,8]^ and does not always prevent hyperglycemia^[9]^, especially when selecting a tight blood glucose target, such as 80-110 mg/dl (4.4-6.1 mmol/l)^[3,4,7,10-12]^. Furthermore, differences in the clinical implications of insulin use among patients with and without diabetes have not been thoroughly examined, since insulin use is typically driven by the blood glucose level without direct regard for diabetic status. To further confound these effects, there is thought to be considerable heterogeneity in insulin use among hospitals performing CABG in similar patient populations; as a result of these data inconsistencies, there is an imminent need for further study of insulin use in this patient population^[5,11-13]^.

In our study, we explore the characteristics of insulin use by examining a large multi-institutional patient repository and we compare clinical outcomes between patients with and without diabetes as a function of insulin use. We also report hospital-level patterns of insulin use following CABG surgery and the degree of variation among these centers.

## METHODS

### Data Sources

The Society of Thoracic Surgeons (STS) Adult Cardiac Surgery Database (ACSD) was established in 1987 as a multicenter data repository for quality improvement and clinical research. The STS ACSD presently collects data from nearly 90% of all hospitals in the United States of America with cardiothoracic surgical programs and contains detailed data including patients’ demographics, clinical profiles, and in-hospital outcomes. Data definitions are standardized, and data coordinators at individual sites receive specific training in data entry and management. Case report forms from participating sites in the United States of America and Canada are submitted to the data coordinating center (Duke Clinical Research Institute, Durham, North Carolina, United States of America) on a semiannual basis. For quality control, the STS ACSD conducts annual on-site data audits for randomly selected database participants. The accuracy of individual data elements has been validated in regional analyses with an agreement rate of more than 95%^[14]^. Overall completeness of procedure reporting and mortality event reporting in patients aged ≥ 65 years has been validated against national Medicare claims files^[15,16]^.

Fifty STS ACSD institutional members were invited to participate in the Contemporary Analysis of Perioperative Cardiovascular Surgical Care (CAPS-Care) Study, based on a track record of high-quality data submission. Of these, 48 obtained institutional review board approval and, therefore, proceeded with data collection. These participants collected data from a total of 55 hospitals. The STS ACSD contained prospectively collected data related to baseline demographics, clinical and operative variables, and prior cardiopulmonary studies, as well as major adverse events during hospitalization and 30 days postoperatively. The CAPS-Care data collection form included variables related to preoperative clinical encounters, intraoperative care, postoperative pharmacologic care, postoperative management, and postoperative clinical events. CAPS-Care data were entered into a computerized database and linked to STS ACSD data via a unique record identification system. The present study is focused on postoperative insulin use, defined as the first 24 hours after ICU arrival.

### Patient Population

A total of 2,390 patients were included in the analysis and each institution contributed an average of 50 patients (range 8 - 60), representing a randomly selected proportion of patients from each center who met inclusion criteria. Patients who underwent elective or urgent CABG from January 2004 to June 2005 were included. “High-risk” patients were defined as those with preoperative ejection fraction < 40% or age ≥ 65 years, with either diabetes mellitus or estimated glomerular filtration rate (eGFR) < 60 mL/min per 1.73 m^2^. Diabetes was defined per the STS database definitions, which includes patients diagnosed and treated by a healthcare provider for diabetes as defined by one of several criteria according to the American Diabetes Association. We excluded patients who were less than 18 years of age, undergoing emergent CABG, or having preoperative cardiogenic shock.

### Statistical Analysis

The total cohort was divided into three groups: 1) patients with diabetes receiving insulin; 2) patients without diabetes receiving insulin; and 3) all patients not receiving insulin. Patient characteristics and comorbidities were compared among groups. Categorical variables are presented as percentages; continuous variables are presented as medians and interquartile ranges, unless otherwise stated. The Kruskal-Wallis test was used for statistical comparison of continuous variables and Mantel-Haenszel chi-square analysis was applied to the remaining categorical variables. Unadjusted complication rates were reported including acute renal failure, new-onset hemodialysis, new-onset atrial fibrillation, reoperation, and perioperative myocardial infarction. Operative mortality was included in our outcomes.

Multivariable logistic regression with generalized estimating equations with a compound symmetric working correlation matrix and empirical (sandwich) standard error estimates were used to determine the association of insulin use with perioperative mortality and incidence of major complications. The variables entered into the model for risk adjustment consisted of male gender, Caucasian race, age, smoker, diabetes status, hypercholesterolemia, hypertension, body mass index, body surface area, cerebrovascular accident (recent and remote), endocarditis (any active or treated), chronic lung disease (mild, moderate, severe), immunosuppressive treatment, peripheral vascular disease, cerebrovascular disease, prior CABG, prior valve surgery, prior intrapericardial or great vessel surgery, prior pacemaker, prior myocardial infarction (within 1 day, 1-7 days, 1-3 weeks, > 3 weeks), arrhythmia, preoperative atrial fibrillation, congestive heart failure, New York Heart Association Class IV, left main or triple vessel disease, aortic stenosis, aortic insufficiency, mitral regurgitation, tricuspid regurgitation, operative status (elective, urgent), cardiogenic shock, prior percutaneous coronary intervention, ejection fraction, dialysis, eGFR, surgery date (6-month intervals; with spring 2004, fall 2004, and spring 2005), and interactions between gender and body mass index. All analyses were conducted using the SAS software, versions 8.2, 9.3, and 9.4 (SAS Institute Inc., Cary, North Carolina, United States of America).

## RESULTS

### Patient Characteristics

The analysis included 2,390 patients undergoing CABG between January 2004 and June 2005. These patients represent 55 hospitals from 48 STS ACSD participating sites. The median number of patients per hospital was 50 (range 8 - 60). There were 1,258 (52.63%) patients with diabetes and 1,132 (47.36%) patients without diabetes.

Of the 2,390 patients, 1,959 (81.96%) received insulin postoperatively. Insulin was administered to 1,203 (50.33%) patients with diabetes and to 756 (31.64%) patients without diabetes. Patients with diabetes that received insulin were slightly younger; had higher body mass index; and had higher rates of dyslipidemia, hypertension, chronic lung disease, and previous stroke. Patients not receiving insulin (12.76% of whom had diabetes) had higher rates of smoking and chronic lung disease but presented with a generally lower New York Heart Association class (Table 1). Patients with diabetes receiving insulin also tended to have higher left ventricular ejection fractions than the comparison groups. Off-pump CABG was also more prevalent among those not receiving insulin (*P*<0.001) (Table 2).

**Table 1:** Patients' characteristics.

	Total (n=2390) (%)	Insulin used, patients without diabetes (n=756) (%)	Insulin used, patients with diabetes (n=1203) (%)	No insulin used (n=431) (%)	*P*-value
Age, median	72.00	73.00	71.00	73.00	<0.001
Male gender	66.40	67.59	63.59	72.16	0.004
BMI, median	28.34	27.14	29.57	27.01	<0.001
BMI ≥ 35	13.10	8.73	17.62	8.12	<0.001
Smoking history	60.93	60.31	58.35	69.14	<0.001
Diabetes					<0.001
Diet-controlled	2.76	0.00	4.32	3.25	
Oral antihyperglycemics	31.55	0.00	60.76	5.34	
Insulin-dependent	16.53	0.00	32.09	2.09	
Family history of CAD	35.27	35.85	33.17	40.14	0.034
Dyslipidemia	75.98	73.15	77.64	76.33	0.060
Preoperative creatinine, median	1.10	1.20	1.10	1.20	<0.001
Renal failure	8.66	7.80	9.48	7.89	0.365
ESRD on dialysis	28.02	23.73	31.58	23.53	0.455
Hypertension	83.89	79.89	87.95	79.58	<0.001
CVA					0.012
Remote	10.84	8.99	12.88	8.35	
Recent	0.50	0.53	0.33	0.93	
Chronic lung disease					0.007
Mild/moderate	22.09	24.21	21.36	20.42	
Severe	5.10	6.88	4.49	3.71	
Peripheral vascular disease	19.92	19.05	19.78	21.81	0.512
Cerebrovascular disease	21.92	21.43	22.86	20.19	0.476
Previous MI	47.66	48.54	46.72	48.72	0.650
CHF	30.96	32.14	31.17	28.31	0.379
Angina	74.44	74.47	74.90	73.09	0.761
Arrhythmia					0.020
Atrial fibrillation/atrial flutter	13.10	16.14	11.47	12.30	
Heart block	1.38	1.19	1.33	1.86	
Sustained VT/VF	2.22	2.38	1.58	3.71	
Resuscitation	0.50	0.26	0.33	1.39	0.015
NYHA class					0.006
I	9.79	7.94	9.64	13.46	
II	20.96	20.77	20.86	21.58	
III	43.97	43.12	44.72	43.39	
IV	24.85	27.65	24.52	20.88	

BMI=body mass index; CAD=coronary artery disease; CHF=congestive heart failure; CVA=cerebrovascular accident; ESRD=end-stage renal disease; MI=myocardial infarction; NYHA=New York Heart Association; VF=ventricular fibrillation; VT=ventricular tachycardia

**Table 2:** Preoperative status and operative features.

	Total (n=2390) (%)	Insulin used, patients without diabetes (n=756) (%)	Insulin used, patients with diabetes (n=1203) (%)	No insulin used (n=431) (%)	*P*-value
Previous CABG	6.90	7.94	6.73	5.57	0.286
Previous valve repair/replacement	1.09	1.46	0.83	1.16	0.431
Previous other cardiac surgery	2.13	2.25	2.00	2.32	0.898
Echocardiography					
EF, median (%)	45.00	40.00	48.00	40.00	<0.001
AS	11.67	13.10	11.89	8.58	0.064
MS	1.67	1.59	1.50	2.32	0.509
AI					<0.001
Trivial/mild	12.39	15.61	10.89	10.90	
Moderate/severe	3.60	5.15	2.58	3.72	
MR					<0.001
Trivial/mild	23.77	23.94	23.94	22.97	
Moderate/severe	12.38	17.19	9.64	11.60	
Urgent procedure	48.79	49.21	46.47	54.52	0.016
Procedure category					<0.001
CABG only	74.69	66.01	78.22	80.05	
AVR+CABG	7.74	8.86	7.40	6.73	
MVR+CABG	1.42	1.98	1.16	1.16	
MVr+CABG	3.05	5.16	2.24	1.62	
Other+CABG	13.10	17.99	10.97	10.44	
Off-pump CABG	15.73	13.10	14.96	22.51	<0.001
IABP	10.08	11.90	9.14	9.51	0.132
Perfusion time, median (min)	110.00	111.00	109.00	105.00	0.179
Cross-clamp time, median (min)	76.00	76.00	75.00	76.00	0.092

AI=aortic insufficiency; AS=aortic stenosis; AVR=aortic valve replacement; CABG=coronary artery bypass graft (surgery); EF=ejection fraction; IABP=intra-aortic balloon pump; MR=mitral regurgitation; MS=mitral stenosis; MVr=mitral valve repair; MVR=mitral valve replacement

### Glycemic Control

Overall, the median maximum intraoperative glucose level was 200 mg/dL (range 161 - 246) and the median minimum level was 120 mg/dL (range 102 - 146). Postoperatively, the median maximum glucose level was 200 mg/dL (range 168 - 237) and the median minimum level was 108 mg/dL (range 88 - 132).

With regard to the method of delivery, insulin was administered to 1,232 of 2,390 (51.55%) patients intraoperatively; among these, 640 (51.94%) received continuous infusion, 390 (31.65%) intravenous boluses, and 208 (16.88%) in various combinations. Postoperatively, 1,959 of 2,390 (81.97%) patients received insulin, including continuous infusion in 1,339 (68.35%), intravenous boluses in 307 (15.67%), subcutaneous in 518 (26.44%), and various combinations in 370 (18.88%) patients. Postoperatively, intravenous boluses of insulin were administered to 12.28% of patients without diabetes and to 13.35% of patients with diabetes (*P*=0.43). Subcutaneous insulin was utilized in 18.46% of patients without diabetes and 24.56% of patients with diabetes (*P*=0.0003).

**Fig. 1:**
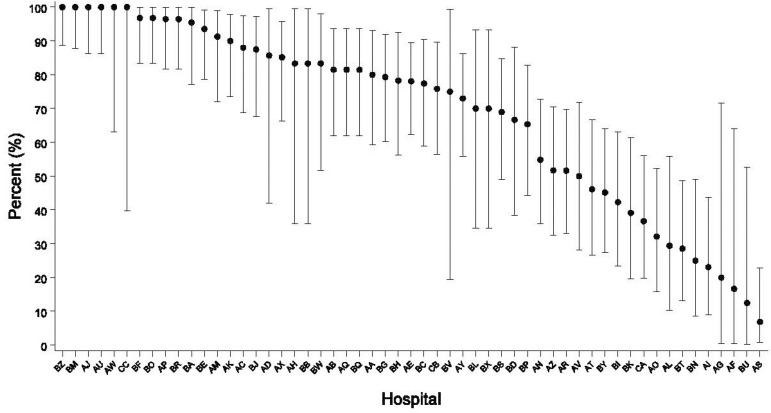
Variation in patients with diabetes. Hospital-by-hospital variation of percentage of continuous insulin infusion used in the first 24 hours in patients with diabetes in the post operating room*.*

**Fig. 2:**
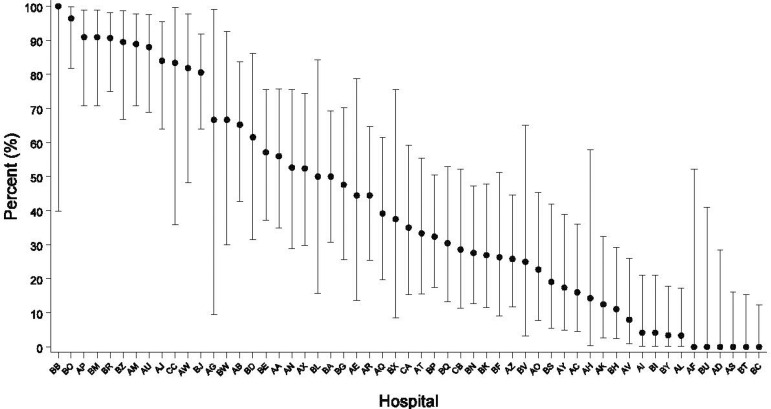
Variation in patients without diabetes. Hospital-by-hospital variation of percentage of continuous insulin infusion used in the first 24 hours in patients without diabetes in the post operating room.

Unadjusted hospital-level usage of postoperative continuous insulin infusion is described in patients with diabetes (Figure 1) and those without it (Figure 2). Only 464 (40.98%) of the patients without diabetes received continuous insulin infusion postoperatively. The rate of continuous insulin infusion in the ICU varied across the range of hospitals. Some hospitals did not use any continuous infusion in patients without diabetes.

### Clinical Outcomes

Overall, the incidence of major complications was the highest in patients without diabetes that received insulin (Table 3). Specifically, rate of reoperation, perioperative myocardial infarction, and prolonged ventilation were significantly higher in this cohort, the latter of which is concordant with a statistically higher incidence of preoperative lung disease in this group. Operative mortality reached 7.28% in patients without diabetes that received insulin, 3.99% in patients with diabetes that received insulin, and 3.71% in the remaining patients that did not receive insulin (*P*=0.002).

**Table 3:** Early morbidity and mortality stratified by insulin use and presence or absence of diabetes mellitus.

	Total (n=2390) (%)	Insulin used, patients without diabetes (n=756) (%)	Insulin used, patients with diabetes (n=1203) (%)	No insulin used (n=431) (%)	*P*-value
In-hospital complications	46.15	50.66	44.89	41.76	0.005
Reoperation	9.33	11.64	8.73	6.96	0.017
Perioperative MI	1.09	1.72	0.75	0.93	0.123
Deep sternal infection	0.54	0.53	0.58	0.46	0.958
Stroke	2.47	3.04	2.66	0.93	0.065
Prolonged ventilation	14.18	16.40	14.46	9.51	0.004
Renal failure	7.66	8.47	7.90	5.57	0.178
New dialysis	2.97	2.65	3.49	2.09	0.276
Atrial fibrillation	25.27	25.40	25.94	23.20	0.609
Operative mortality	4.98	7.28	3.99	3.71	0.002
In-hospital mortality	4.56	6.75	3.66	3.25	0.002
Total length of stay, median (days)	9.00	9.00	9.00	8.00	0.036

MI=myocardial infarction

The unadjusted mortality rate for patients without diabetes receiving postoperative insulin was higher than that of all patients not receiving insulin (odds ratio [OR]=2.03; 95% confidence interval [CI] 1.15-3.57; *P*=0.014). In contrast, patients with diabetes that received insulin had a similar unadjusted mortality rate as those not receiving insulin (OR=1.08; 95% CI 0.62 - 1.88; *P*=0.765). After multivariable risk-adjustment, neither of these comparisons achieved a statistically significant difference with respect to operative mortality. With regard to composite mortality or major complication, both patients with diabetes (OR=1.79; 95% CI 1.36 - 2.35; *P*<0.001) and without it (OR=1.39; 95% CI 1.10-1.75; *P*=0.006) had higher unadjusted rates if they had received postoperative insulin. Subsequent risk-adjusted analysis revealed that insulin use in patients without diabetes was again associated with an increased combined incidence of complications and mortality when compared with those not receiving insulin (OR=1.54; 95% CI 1.15-2.04; *P*=0.003) (Table 4). In contrast, patients with diabetes that received insulin had similar adjusted combined rates of mortality and major complications as those not receiving insulin (adjusted OR=1.01; 95% CI 0.52-1.98; *P*=0.975).

**Table 4:** Risk-adjusted clinical outcomes among 2,390 cardiac surgery patients with and without diabetes receiving perioperative insulin.

	Total (n=2390)	Unadjusted OR	Lower 95% CI	Upper 95% CI	*P*-value
Operative mortality	2390				0.013
Insulin, without diabetes	756	2.03	1.15	3.57	0.014
Insulin, with diabetes	1203	1.08	0.62	1.88	0.795
Composite mortality or major complication	2383				0.002
Insulin, without diabetes	755	1.79	1.36	2.35	<0.001
Insulin, with diabetes	1199	1.39	1.10	1.75	0.006
		**Adjusted** **OR**	**Lower** **95% CI**	**Upper** **95% CI**	***P*-value**
Operative mortality					0.414
Insulin, without diabetes		1.47	0.82	2.62	0.194
Insulin, with diabetes		1.28	0.32	5.04	0.727
Composite mortality or major complication					0.030
Insulin, without diabetes		1.54	1.15	2.04	0.003
Insulin, with diabetes		1.01	0.52	1.98	0.975

CI=confidence interval; OR=odds ratio

## DISCUSSION

Insulin use, particularly continuous insulin infusion use, is common in both patients with and without diabetes following CABG. A wide range of variability exists in patterns of insulin use among several participating centers. Despite the clinical benefits of moderate glycemic control in the perioperative setting, insulin use in patients without diabetes was associated with worse clinical outcomes compared to patients (both with and without diabetes) who did not receive insulin in a risk-adjusted model.

After years of strict glucose control with intensive insulin regimens, multiple quality studies have been unable to reproduce the findings presented by Van den Berghe et al. in 2001^[3,9,13]^. In 2006, Van den Berghe et al.^[11]^ demonstrated that among 433 patients admitted to the ICU for less than three days, intensive insulin therapy was related to greater mortality. Furthermore, the Nice-Sugar Study reported increased mortality (27.5%) at 90 days with intensive glucose control compared to conventional control (24.9%; OR=1.14, 95% CI 1.02-1.28; *P*=0.02). Severe hypoglycemia was also found to occur more often with intensive insulin management (6.8% *vs.* 0.5%, *P*<0.001)^[7]^. In addition to the establishment of these appropriate target ranges, actual achievement of glycemic control can be clinically challenging. These studies, with varying designs, were also limited by not taking into account the variation in phlebotomy sites (*i.e.*, arterial, venous, or capillary), enteral *vs.* parenteral diet, and specific insulin use patterns^[13,17,18]^. Therefore, it is evident that the optimal method of glycemic control may vary among different subgroups of patients, and no single protocol is definitively appropriate for all patients after cardiac surgery.

In the operating room, insulin is almost always delivered intravenously (bolus or continuous) given the more immediate effect and predictable response compared to subcutaneous injections. In contrast, subcutaneous and bolus methods tend to be preferred in patients without diabetes in the postoperative setting. In the existing published literature, insulin administration methods vary widely in terms of clinical protocols and adjustment scales, and our results reiterate this institutional variability. In our study, insulin use in patients without diabetes is associated with higher morbidity and mortality rates compared to patients with diabetes. While the postoperative maximum blood glucose level has been shown to be an independent predictor of mortality in patients without diabetes, hyperglycemia is not associated with higher adjusted mortality in patients with diabetes^[19]^. These results emphasize the need to stratify guidelines for glycemic control by different subgroups of patients, particularly on the basis of baseline diabetes status.

The mechanisms of glucose variation around the time of CABG surgery are not completely understood. While acute myocardial injury or inflammation related to cardiopulmonary bypass may result in insulin resistance and subsequent hyperglycemia, often called “diabetes of injury”, alternative mechanisms have yet to be identified^[20]^. Furthermore, the association between acute illness and hyperglycemia remains unclear as far as whether hyperglycemia is a marker of multisystem stress *vs.* a mediator of complications. Regardless, hyperglycemia has been associated with poor clinical outcomes in critically ill patients^[4,21]^. Taking into account the role of hyperglycemia as a marker of stress during and after cardiac surgery, it is feasible that insulin administration in this setting is blunting other necessary physiologic pathways leading to worse clinical outcomes. Given the physiologic effects of insulin that extend well beyond glycemic control, perhaps the anabolic effects of this hormone are poorly tolerated in the immediate postoperative period. Further investigation is warranted to determine the pathways by which insulin is counterproductive to patient recovery and clinical outcomes.

Additional patient and procedural characteristics may also have factored into the results observed in this study. For example, preoperative lung disease and arrhythmias were more prevalent in non-diabetic patients receiving insulin. This likely has a direct influence on patient outcomes, though multivariable adjustment did not demonstrate this to be a significant contributor to our clinical endpoints. The intraoperative course also is likely to play a role, with particular attention to concomitant valve surgery, which occurred more frequently in those who did not receive insulin. The causation between concomitant procedures and outcomes are not fully understood, but additional bypass time required for these procedures may incur a greater systemic response that, in turn, requires more insulin use. Better understanding the interactions between these elements will be useful in future studies.

### Limitations

Our study had several limitations. First, despite the prospective method of data collection used in the CAPS-Care database, we were limited by its post hoc design; however, we were able to perform robust adjusted analyses for several covariates among a well-powered cohort. Second, we recognize the large degree of variability among centers in relation to intraoperative and postoperative blood glucose management, as well as overall intensive care strategies. In fact, several individual patients had a combination of insulin administration routes and strategies both during and after surgery, which further adds to this limitation. This variability is likely to represent differences in practice behaviors, which may confound the indications for insulin administration at individual centers. Furthermore, the study cohort arises from patients undergoing surgery between 2004 and 2005, which may reflect practices that are less generalizable to today’s standards. However, in the current era, there still remains a lack of definitive evidence regarding the appropriate use of insulin in this setting, as well as a lack of insight into the effects of insulin use in patients without diabetes - both of which were found to be significant in our study. This further emphasizes the importance of standardizing postoperative glycemic protocols at any given institution. Finally, the choice of insulin adjustment scale and insulin formulation are among several factors that vary among participating institutions. As a limitation of the database utilized, we were not able to account for these variations, any of which may impact the adequacy of postoperative glucose management.

## CONCLUSION

Insulin use and blood glucose management strategies vary greatly among centers performing CABG in both patients with and without diabetes. Overall, patients without diabetes receiving postoperative insulin appear to have higher mortality and more complications compared with patients not receiving insulin. The mechanisms behind these trends remain unclear and deserve further investigation in order to optimize postoperative glycemic control and mitigate the sequelae of postoperative hyperglycemia, particularly in patients without diabetes.

**Table:** 

Authors' roles & responsibilities	
DNR	Substantial contributions to the conception or design of the work; or the acquisition, analysis, or interpretation of data for the work; drafting the work or revising it critically for important intellectual content; final approval of the version to be published; agreement to be accountable for all aspects of the work in ensuring that questions related to the accuracy or integrity of any part of the work are appropriately investigated and resolved
JBW	Substantial contributions to the conception or design of the work; or the acquisition, analysis, or interpretation of data for the work; drafting the work or revising it critically for important intellectual content; final approval of the version to be published; agreement to be accountable for all aspects of the work in ensuring that questions related to the accuracy or integrity of any part of the work are appropriately investigated and resolved
ASA	Substantial contributions to the conception or design of the work; or the acquisition, analysis, or interpretation of data for the work; drafting the work or revising it critically for important intellectual content; final approval of the version to be published; agreement to be accountable for all aspects of the work in ensuring that questions related to the accuracy or integrity of any part of the work are appropriately investigated and resolved
SL	Substantial contributions to the conception or design of the work; or the acquisition, analysis, or interpretation of data for the work; drafting the work or revising it critically for important intellectual content; final approval of the version to be published; agreement to be accountable for all aspects of the work in ensuring that questions related to the accuracy or integrity of any part of the work are appropriately investigated and resolved
RAKK	Substantial contributions to the conception or design of the work; or the acquisition, analysis, or interpretation of data for the work; drafting the work or revising it critically for important intellectual content; final approval of the version to be published; agreement to be accountable for all aspects of the work in ensuring that questions related to the accuracy or integrity of any part of the work are appropriately investigated and resolved
EDP	Substantial contributions to the conception or design of the work; or the acquisition, analysis, or interpretation of data for the work; drafting the work or revising it critically for important intellectual content; final approval of the version to be published; agreement to be accountable for all aspects of the work in ensuring that questions related to the accuracy or integrity of any part of the work are appropriately investigated and resolved
RDL	Substantial contributions to the conception or design of the work; or the acquisition, analysis, or interpretation of data for the work; drafting the work or revising it critically for important intellectual content; final approval of the version to be published; agreement to be accountable for all aspects of the work in ensuring that questions related to the accuracy or integrity of any part of the work are appropriately investigated and resolved

## References

[1] Yusuf S, Zucker D, Peduzzi P, Fisher LD, Takaro T, Kennedy JW (1994). Effect of coronary artery bypass graft surgery on survival: overview of 10-year results from randomised trials by the coronary artery bypass graft surgery trialists collaboration. Lancet.

[2] ElBardissi AW, Aranki SF, Sheng S, O’Brien SM, Greenberg CC, Gammie JS (2012). Trends in isolated coronary artery bypass grafting: an analysis of the society of thoracic surgeons adult cardiac surgery database. J Thorac Cardiovasc Surg.

[3] van den Berghe G, Wouters P, Weekers F, Verwaest C, Bruyninckx F, Schetz M (2001). Intensive insulin therapy in critically ill patients. N Engl J Med.

[4] Moghissi ES, Korytkowski MT, DiNardo M, Einhorn D, Hellman R, Hirsch IB (2009). American association of clinical endocrinologists and American diabetes association consensus statement on inpatient glycemic control. Diabetes Care.

[5] Wiener RS, Wiener DC, Larson RJ (2008). Benefits and risks of tight glucose control in critically ill adults: a meta-analysis. JAMA.

[6] Dellinger RP, Levy MM, Carlet JM, Bion J, Parker MM, Jaeschke R (2008). Surviving sepsis campaign: international guidelines for management of severe sepsis and septic shock: 2008. Intensive Care Med.

[7] Finfer S, Chittock DR, Su SY, Blair D, Foster D, Dhingra V, NICE-SUGAR Study Investigators (2009). Intensive versus conventional glucose control in critically ill patients. N Engl J Med.

[8] Chaney MA, Nikolov MP, Blakeman BP, Bakhos M (1999). Attempting to maintain normoglycemia during cardiopulmonary bypass with insulin may initiate postoperative hypoglycemia. Anesth Analg.

[9] Butterworth J, Wagenknecht LE, Legault C, Zaccaro DJ, Kon ND, Hammon JW Jr (2005). Attempted control of hyperglycemia during cardiopulmonary bypass fails to improve neurologic or neurobehavioral outcomes in patients without diabetes mellitus undergoing coronary artery bypass grafting. J Thorac Cardiovasc Surg.

[10] Lazar HL, McDonnell M, Chipkin SR, Furnary AP, Engelman RM, Sadhu AR (2009). The society of thoracic surgeons practice guideline series: blood glucose management during adult cardiac surgery. Ann Thorac Surg.

[11] Van den Berghe G, Wilmer A, Hermans G, Meersseman W, Wouters PJ, Milants I (2006). Intensive insulin therapy in the medical ICU. N Engl J Med.

[12] Gandhi GY, Nuttall GA, Abel MD, Mullany CJ, Schaff HV, O’Brien PC (2007). Intensive intraoperative insulin therapy versus conventional glucose management during cardiac surgery: a randomized trial. Ann Intern Med.

[13] Griesdale DE, de Souza RJ, van Dam RM, Heyland DK, Cook DJ, Malhotra A (2009). Intensive insulin therapy and mortality among critically ill patients: a meta-analysis including NICE-SUGAR study data. CMAJ.

[14] Welke KF, Ferguson TB Jr, Coombs LP, Dokholyan RS, Murray CJ, Schrader MA (2004). Validity of the society of thoracic surgeons national adult cardiac surgery database. Ann Thorac Surg.

[15] Ferguson TB Jr, Peterson ED, Coombs LP, Eiken MC, Carey ML, Grover FL (2003). Use of continuous quality improvement to increase use of process measures in patients undergoing coronary artery bypass graft surgery: a randomized controlled trial. JAMA.

[16] Welke KF, Peterson ED, Vaughan-Sarrazin MS, O’Brien SM, Rosenthal GE, Shook GJ (2007). Comparison of cardiac surgery volumes and mortality rates between the society of thoracic surgeons and medicare databases from 1993 through 2001. Ann Thorac Surg.

[17] Inzucchi SE, Siegel MD (2009). Glucose control in the ICU--how tight is too tight?. N Engl J Med.

[18] Van den Berghe G, Schetz M, Vlasselaers D, Hermans G, Wilmer A, Bouillon R (2009). Clinical review: intensive insulin therapy in critically ill patients: NICE-SUGAR or Leuven blood glucose target?. J Clin Endocrinol Metab.

[19] Van den Berghe G (2004). How does blood glucose control with insulin save lives in intensive care?. J Clin Invest.

[20] Bellodi G, Manicardi V, Malavasi V, Veneri L, Bernini G, Bossini P (1989). Hyperglycemia and prognosis of acute myocardial infarction in patients without diabetes mellitus. Am J Cardiol.

[21] Székely A, Levin J, Miao Y, Tudor IC, Vuylsteke A, Ofner P (2011). Impact of hyperglycemia on perioperative mortality after coronary artery bypass graft surgery. J Thorac Cardiovasc Surg.

